# A Genomic Perspective on the Potential of Wild-Type Rumen Bacterium *Enterobacter* sp. LU1 as an Industrial Platform for Bio-Based Succinate Production

**DOI:** 10.3390/ijms21144835

**Published:** 2020-07-08

**Authors:** Hubert Szczerba, Karolina Dudziak, Mariusz Krawczyk, Zdzisław Targoński

**Affiliations:** 1 Department of Biotechnology, Microbiology and Human Nutrition, University of Life Sciences in Lublin, 20-704 Lublin, Poland; zdzislaw.targonski@up.lublin.pl; 2 Chair and Department of Biochemistry and Molecular Biology, Medical University of Lublin, 20-093 Lublin, Poland; karolina.dudziak23@gmail.com; 3 Genomed SA, 02-971 Warsaw, Poland; mkraw@genomed.pl

**Keywords:** complete genome sequence, *Enterobacter*, glycerol, succinic acid production

## Abstract

*Enterobacter* sp. LU1, a wild-type bacterium originating from goat rumen, proved to be a potential succinic acid producer in previous studies. Here, the first complete genome of this strain was obtained and analyzed from a biotechnological perspective. A hybrid sequencing approach combining short (Illumina MiSeq) and long (ONT MinION) reads allowed us to obtain a single continuous chromosome 4,636,526 bp in size, with an average 55.6% GC content that lacked plasmids. A total of 4425 genes, including 4283 protein-coding genes, 25 ribosomal RNA (rRNA)-, 84 transfer RNA (tRNA)-, and 5 non-coding RNA (ncRNA)-encoding genes and 49 pseudogenes, were predicted. It has been shown that genes involved in transport and metabolism of carbohydrates and amino acids and the transcription process constitute the major group of genes, according to the Clusters of Orthologous Groups of proteins (COGs) database. The genetic ability of the LU1 strain to metabolize a wide range of industrially relevant carbon sources has been confirmed. The genome exploration indicated that *Enterobacter* sp. LU1 possesses all genes that encode the enzymes involved in the glycerol metabolism pathway. It has also been shown that succinate can be produced as an end product of fermentation via the reductive branch of the tricarboxylic acid cycle (TCA) and the glyoxylate pathway. The transport system involved in succinate excretion into the growth medium and the genes involved in the response to osmotic and oxidative stress have also been recognized. Furthermore, three intact prophage regions ~70.3 kb, ~20.9 kb, and ~49.8 kb in length, 45 genomic islands (GIs), and two clustered regularly interspaced short palindromic repeats (CRISPR) were recognized in the genome. Sequencing and genome analysis of *Enterobacter* sp. LU1 confirms many earlier results based on physiological experiments and provides insight into their genetic background. All of these findings illustrate that the LU1 strain has great potential to be an efficient platform for bio-based succinate production.

## 1. Introduction

*Enterobacter* sp. LU1 is a Gram-negative, rod-shaped, wild-type bacterium that has been isolated from goat rumen by bacterial enrichment and selective culture for succinic acid (SA)-producing bacteria [1]. SA, with a molecular formula of C_4_H_6_O_4_, is natural organic acid that exists in animals, plants, and microorganisms [2]. It has been recognized as one of the top 10 most promising C4-chemical building blocks with prospects for bio-based commercial production [3,4]. SA can be converted into industrially relevant chemicals, including adipic acid, 1,4-butanediol (1,4-BDO), tetrahydrofuran (THF), γ-butyrolactone (GBL), N-methylopyrrolidone (NMP), and 2-pyrrolidone [5]. Nowadays, succinate is also used as an additive in food, agriculture, and pharmaceutical industries as well as in biodegradable polymer production, including polybutylene succinate (PBS) and polyamides [6,7].

To date, various chemical routes have been developed for SA production, including chemical synthesis from *n*-butane through maleic anhydrate, the most commonly used method in commercial succinate production [8]. However, depletion of fossil fuels and our ever-increasing concern about environmental pollution urge us to establish sustainable processes for bio-based production of high-valuable commodity and specialty chemicals from waste feedstock. Compared with petrochemical synthesis, biotechnological production of SA is characterized by high efficiency, low cost, and renewability of substrates [2]. Therefore, a sustainable, ecofriendly process for microbial production of SA from renewable feedstock has become a focal point of global interest [9,10,11,12].

*Actinobacillus succinogenes* and *Anaerobiospirillum succiniciproducens* are considered to be promising SA biocatalysts for industrial application due to their high production efficiency [2]. *Basfia succiniciproducens* and *Mannheimia succiniciproducens* are other microorganisms that have been recognized as native succinate producers [13,14]. Many studies on the physiological and biochemical features of these bacteria have already been carried out. In addition, genomes of these microorganisms have been sequenced, and genetic characterization was performed, facilitating further genetic modifications. Meanwhile, knowledge about genetic features of *Enterobacter* strains, constituting a new source of native biocatalysts for efficient succinate production, is still limited.

In our previous studies, identification and physiological characterization of *Enterobacter* sp. LU1 were carried out [1]. The strain has been shown to be able to efficiently produce SA utilizing valuable feedstocks, specifically crude glycerol and whey permeate derived from the petroleum and whey industries, respectively [15]. Under anaerobic conditions, succinate concentration in culture medium reached 69 g/L, while the final titer of SA under microaerophilic conditions on glycerol alone was 37 g/L [15]. In the present study, a complete genome sequence of *Enterobacter* sp. LU1 was obtained and genetic characterization was carried out as the next step toward better understanding the unique properties of this strain.

Genome sequencing was recognized as an efficient approach for investigation of gene functions [16,17]. Due to the potential use of *Enterobacter* sp. LU1 as an industrial strain, functional analysis of the genome, including identification of genes involved in SA biosynthesis pathways, should be performed. The complete genome sequence will allow for phylogenomic analysis, ensuring accurate and reliable strain identification and facilitating further comparative studies. Furthermore, it will provide insight into the genetic background of several important physiological traits, such as the ability to metabolize a wide range of carbon sources, the capacity to grow under both aerobic and anaerobic conditions, and resistance to high osmotic pressure.

Many genomes of microorganisms that have been recognized as potential producers of bulk chemicals were obtained [17]. Nevertheless, the vast majority are draft genomes that have been sequenced mainly using short read technologies, such as 454 Life Sciences or Illumina [18,19,20]. As a result, many genomes are incomplete multicontig builds, which hinders both complete genome assembly and comparative genomic studies [17]. Therefore, due to the limited reading length, the short read-based sequencing approach using the Illumina platform is not optimal for the *de novo* assembly of whole genome. However, because of the high reading accuracy, this platform is suitable for guided assembly and read mapping combined with long read technology (Oxford Nanopore Technologies, ONT) [21]. The combination of these two platforms creates a robust pipeline, which ensures highly contiguous assembly and high consensus accuracy, making accurate gene identification and characterization possible.

Here, we report the first complete genome sequence and detailed genomic analysis of *Enterobacter* sp. LU1 established by highly efficient and cost-effective hybrid sequencing combining long read single-molecule nanopore sequencing by ONT MinION with the short read Illumina MiSeq platform. The results suggest that this wild-type strain has the genetic background to be an efficient succinate producer. 

## 2. Results and Discussion

### 2.1. Phylogenomic Classification of Enterobacter sp. LU1

From the perspective of studying the LU1 strain as a potential platform for the industrial production of bulk chemicals, determining of its accurate taxonomic position is crucial. In the previous study, the LU1 strain was first identified as *Enterobacter cloacae* based on the 16S rDNA sequence (National Center for Biotechnology Information (NCBI) accession number: KU499554) and phylogenetic analysis. However, analytical profile index (API) testing indicated that the strain showed greater biochemical similarity to *Enterobacter hormaechei* subsp. s*teigerwaltii* than *E. cloacae*, due to its ability to utilize d-arabitol or d-adonitol, for example [1]. Finally, strain LU1 was assigned to *Enterobacter cloacae* complex (ECC), a diverse and closely related bacterial species group to which both species belong. 

**Figure 1:**
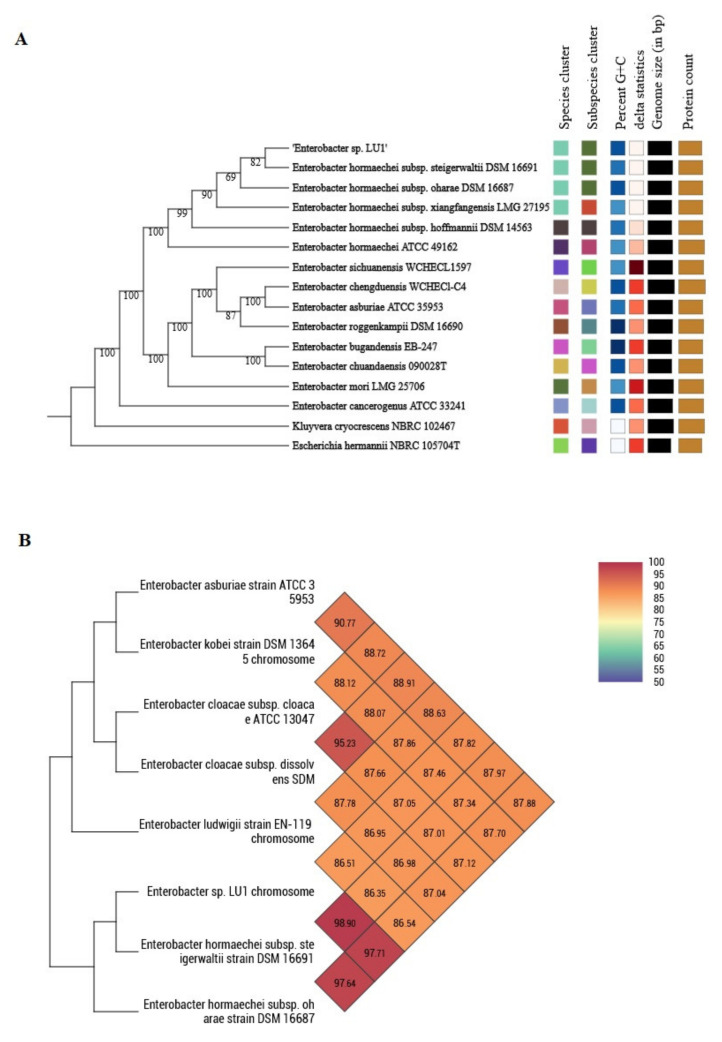
Phylogenomic classification of *Enterobacter* sp. LU1 strain based on: (**A**) Genome Basic Local Alignment Search Tool (BLAST) distance phylogenies (GBDP) using Type Strain Genome Server (TYGS) platform [23]. Tree was inferred with FastME 2.1.6.1 [29] from GBDP distances calculated from genome sequences. Branch lengths are scaled in terms of GBDP distance formula *d*_5_. Numbers above branches are GBDP pseudo-bootstrap support values > 60% from 100 replications, with an average branch support of 94.4%. Tree was rooted at midpoint [30]. (**B**) OrthoANI values using Orthologous Average Nucleotide Identity Tool (OAT) software (https://www.ezbiocloud.net/tools/orthoani) [27]. Heatmap presents OrthoANI values of *Enterobacter* sp. LU1 and seven closely related *Enterobacter* species belonging to *Enterobacter cloacae* complex (ECC).

Because of the polyphyletic nature of the genus *Enterobacter*, it has been proven that accurate identification of ECC isolates at the species level often becomes problematic due to imprecise taxonomy and failure of the standard identification methods [22]. Hence, to determine the accurate and reliable taxonomic position of LU1 strain, whole-genome-based taxonomic analysis was undertaken with the Type Strain Genome Server (TYGS) platform [23] (Figure 1A) [24]. The TYGS results show that LU1 strain is most closely related to *E. hormaechei* subsp. *steigerwaltii*, which confirmed previous API-based identification [1]. A low δ value of 0.137 was observed, which corresponds to high average branch support for generated tree and high phylogenetic accuracy [25]. The digital DNA–DNA hybridization (dDDH) of LU1 strain with two best Basic Local Alignment Search Tool (BLAST) hits, namely, *E. hormaechei* subsp. *steigerwaltii* DSM 16691 and *Enterobacter hormaechei* subsp. *oharae* DSM 16687 strains, was 93.2% and 89.1%, with guanine-cytosine (GC) content difference of 0.07% and 0.04%, respectively. Among the methods for evolutionary distance assessment between bacterial species based on digital whole genome comparison, average nucleotide identity (ANI) is one of the most powerful measurements [26]. Therefore, to make identification more accurate, eight complete genomes of closely related *Enterobacter* species belonging to ECC, including the LU1 strain genome, were retrieved from NCBI GenBank database and Orthologous Average Nucleotide Identity Tool (OAT) software and their relationships and evolutionary distance were assessed based on OrthoANI values [27]. As shown in Figure 1B, all of ANI values are high (> 86.35%), which confirms the close relationship between ECC strains. LU1 strain exhibited the highest OrthoANI value of 98.9% with *E. hormaechei* subsp. *steigerwaltii* DSM 16691, and 97.71% with *E. hormaechei* subsp. *oharae*, both above the species boundary value (ANI > 95–96%), confirming the results obtained by TYGS analysis [25,27]. In turn, the OrthoANI value of LU1 strain with *Enterobacter cloacae* subsp. *cloacae* ATCC 13047 and subsp. *dissolvens* SDM was 87.05% and 86.95%, respectively. These results clearly indicate that *Enterobacter* sp. LU1 should be classified as an *E. hormaechei* species and more specifically as the *E. hormaechei* subsp. *steigerwaltii* strain [28].

### 2.2. Assembly, Structure, and General Features of Enterobacter sp. LU1 Genome

**Figure 2:**
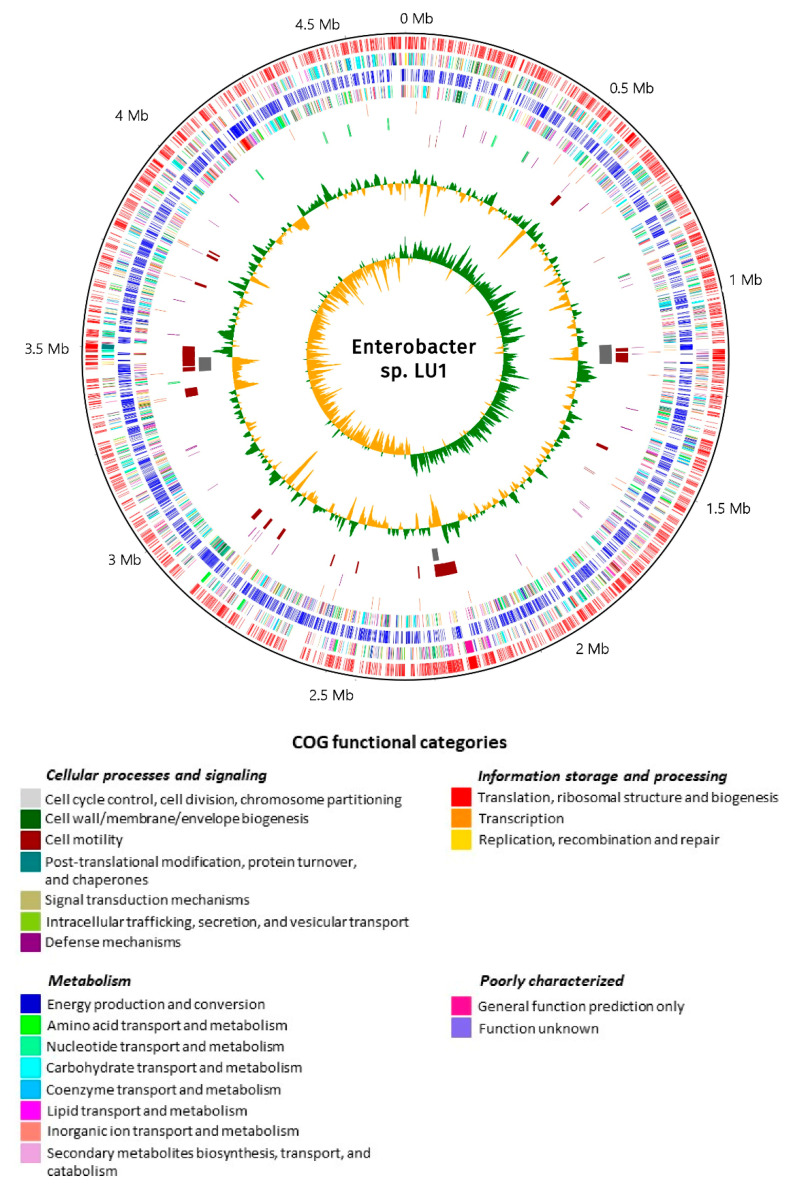
Circular map of *Enterobacter* sp. LU1 genome. From the outer to the inner circle, representation is as follows: 1: position in megabases (black); 2: forward strand coding sequences (CDSs) (red); 3: forward strand clusters of orthologous groups (COGs) (legend); 4: reverse strand CDSs (blue); 5: reverse strand COGs (legend); 6: pseudogenes (dark orange); 7: ribosomal RNAs (rRNAs) (lime) and transfer RNAs (tRNAs) (purple); 8: horizontal gene transfer (HGT) regions (dark red); 9: phage sequences (gray); 10: GC content (green and orange correspond to higher and lower than average content, respectively); 11: GC skew (green and orange correspond to higher and lower than average skew, respectively). Whole-genome sequence visualization was performed using CGView software [33].

The complete genome of *Enterobacter* sp. LU1 was obtained by combining the assembly of data from ONT MinION and Illumina MiSeq sequencing platforms (Table 1). In brief, the standard short insert library of about 400 bp yielded 457 million (mln) bases (2,154,294 paired reads of 250 bases in length) forming 58 contigs with about 98-fold sequencing depth for Illumina MiSeq data. In turn, the MinION sequencer yielded 749 mln bases (54,161 reads) with about 150-fold average coverage. Finally, de novo sequence data assembly from both sequencing platforms was carried out using Unicycler v. 0.4.7 software [31]. Application of third (ONT MinION) and second (Illumina MiSeq) generation sequencing enabled full assembly of the genome and provided high accuracy. Consequently, one single continuous chromosome (NCBI accession number: CP043438) of 4,636,526 bp in size, with an average 55.6% GC content and no plasmids, was obtained (Figure 2). In total, 4425 genes have been annotated in the LU1 strain genome, of which 4283 genes were assigned as putative protein-coding genes. In addition, 114 (2.58%) RNA-encoding genes, including 25 for rRNAs, 84 for tRNAs, and 5 for ncRNAs, and 49 pseudogenes were also predicted in the genome (Table 2). Importantly, the identified tRNA-encoding genes correspond to all 20 standard amino acids: Leu (eight tRNA-encoding genes); Arg (7); Met, Val, and Gly (6); Ala, Thr, Ser, and Lys (5); Asn, Gln, Ile, and Glu (4); Pro, Asp, and Tyr (3); Phe (2); and His, Trp, and Cys (1). Furthermore, one tRNA-encoding gene for selenocysteine (Sec) was also recognized in the genome (Additional file 1). The general properties of LU1 strain genome compared to the genomes of another SA-producer *Klebsiella aerogenes* LU2 (formerly *Enterobacter aerogenes*) and 15 other *Enterobacteriaceae* strains that were retrieved from the NCBI GenBank database are summarized in Table 2. The genome size, total number of genes, and predicted protein-coding genes of LU1 strain are smaller compared to both LU2 strain and the average of 15 *Enterobacteriaceae* strains. However, LU1 strain has slightly larger GC content and the most rRNA-encoding genes (Table 2) (Supplementary Table S1). According to Trujillo et al. [32], a large number of rRNA-encoding genes allow for rapid response to the changing availability of nutrients. This is particularly important in complex ecological niches such as rumen, where strong competition among microorganisms is observed [17]. Compared to other strains gathered in Table 2, *Enterobacter* sp. LU1 has the smallest number of pseudogenes, constituting only 1.11% of all predicted genes. The average gene length for LU1 strain genome was 1047.8 bp and the total length of protein-coding genes amounted to 4,487,738 bp, which constitutes 96.79% of the whole genome sequence length. 

### 2.3. COG Classification of Predicted Genes

**Figure 3:**
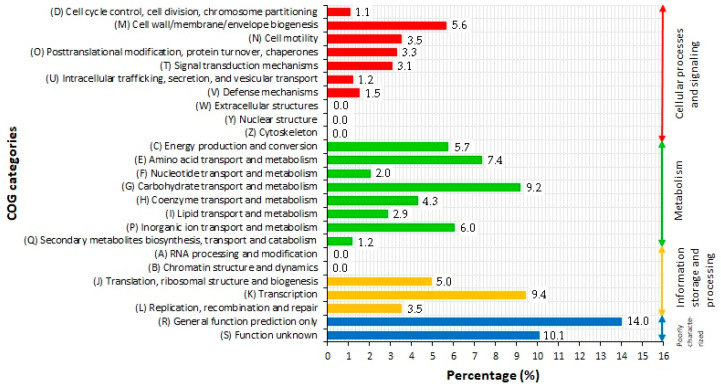
Classification of COG functional annotation of *Enterobacter* sp. LU1. Colored bars indicate percentage of genes assigned to each COG category.

The COG database, a tool for genome-scale phylogenetic classification of encoded proteins, was used for functional studies of predicted genes, whose distribution within the COG categories is provided in Figure 1 and Figure 3, and Additional file 1 [17,34]. A total of 3707 of 4283 (86.55%) coding sequences were assigned to 20 out of 25 COG functional categories belonging to four functional classes: cellular process and signaling (eight categories), metabolism (seven categories), information storage and processing (three categories), and poorly characterized (two categories). The metabolism class was found to be the largest functional group, which has been assigned 1435 genes, constituting 38.7% of all predicted coding sequences, followed by poorly characterized class (892; 24.1%), cellular process and signaling class (717; 19.3%), and information storage and processing class (663; 17.9%). The analysis of gene distribution within COG categories revealed the main functional gene cluster, which included genes involved in carbohydrate transport and metabolism (G), amino acid transport and metabolism (E), and transcription (K) process, collectively constituting 26% of all predicted coding sequences (CDSs). The high proportion of genes belonging to the abovementioned categories suggest that LU1 strain has a well-developed system for the transport and metabolism of a wide spectrum of C- and N- sources, which is an essential feature for industrial producers of bulk chemicals [17]. Furthermore, the high percentage of genes involved in the transcription process ensure the high metabolic activity of the LU1 strain. As we have shown in our previous studies, genes belonging to G, E, and K categories constitute the largest group of genes for another SA-producer, *K. aerogenes* LU2, which has also been isolated from the rumen [17]. Hence, these findings indicate that microorganisms originating from complex ecological niches may have a genetic predisposition for the transport and metabolism of a wide spectrum of C- and N- sources, which would explain the fact that the rumen was the original source of many well-known bacterial producers of bulk chemicals [1,13].

Another high percentage cluster represented genes involved in inorganic ion transport and metabolism (P), energy production and conversion (C), and cell wall/membrane/envelope biogenesis, constituting 17.3% of all annotated genes. In turn, no genes were assigned to five COG categories: RNA processing and modification (A), chromatin structure and dynamics (B), extracellular structures (W), nuclear structure (Y), and cytoskeleton (Z). Genes belonging to these categories were also not identified in the genomes of *Enterobacter* sp. SA187 and *K. aerogenes* LU2 [17,35]. More than 24% of the predicted CDSs are poorly characterized, including 519 (14%) genes assigned to general prediction function only (R), a category containing genes associated with basic metabolism and physiological functions, and 373 (10.1%) genes assigned to the S category gathering genes with unknown functions.

### 2.4. Gene Ontology Term Annotation

**Figure 4:**
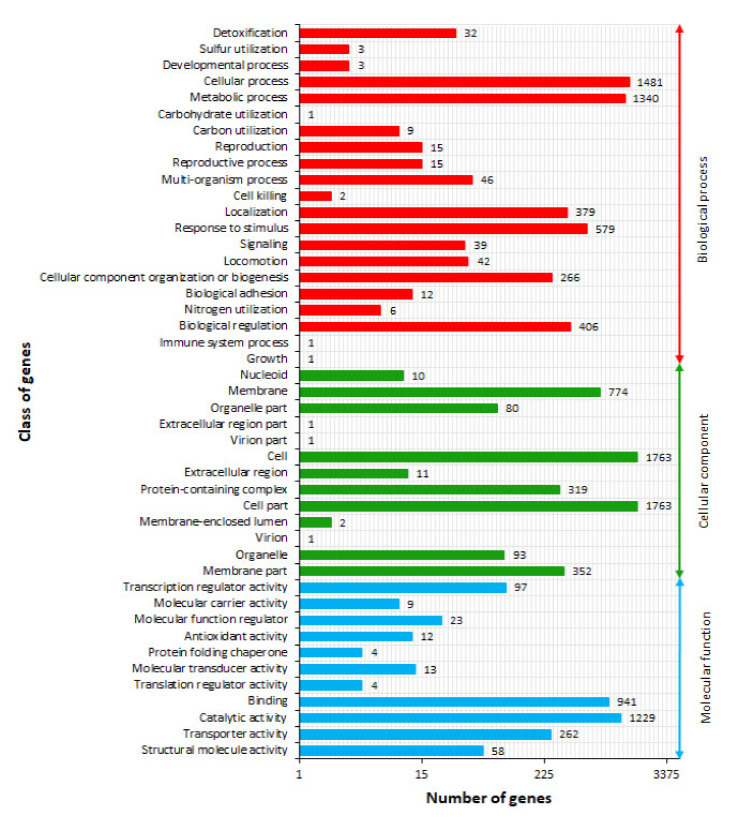
Classification of Gene Ontology (GO) functional annotation of *Enterobacter* sp. LU1. Colored bars indicate numbers of genes assigned to COG categories.

Gene Ontology (GO) annotation is functional classification of predicted genes according to a unified international vocabulary using so-called GO terms, which are helpful in gene associations within particular classes, such as (1) biological process (BP), (2) molecular function (MF), and (3) cellular component (CC) [36]. Therefore, to understand and explain the functional relevance of the LU1 strain genome and classified proteins, GO was investigated and predicted genes were categorized into those three classes by matching them with known sequences. A total of 12,500 genes were assigned to 45 subclasses: 21 subclasses of the BP class, 13 subclasses of the CC class, and 11 subclasses of the MF class, at level 1 (Figure 4) (Additional file 1). The CC class contained the most genes (5170; 41.4%), followed by BP class (4678; 37.4%) and MF class (2652; 21.2%). Within the BP class, metabolic process and cellular process subclasses had 1340 and 1481 genes, respectively, and were the most abundant functional groups, representing 60.3% of genes belonging to the BP class and 22.6% of all assigned genes. In turn, cell and cell part subclasses were recognized as the main functional groups of genes belonging to the CC class, constituting as much as 68.2% of all genes belonging to this class and 28.2% of all assigned genes. Interestingly, it has been shown that the proportions of genes belonging to cell and cell part as well as cellular processes subclasses for *K. aerogenes* LU2 are very close and balanced compared to LU1 strain, indicating that these subclasses are essential and crucial [17]. This also confirms the results of the COG analysis and the fact that the genes involved in primary metabolism of the cell are the largest functional group and present a high degree of evolutionary conservation [37]. Among all subclasses belonging to the MF class, catalytic activity and binding were the most abundant, accounting for 81.8% of GO terms classified to the class and 17.4% of all assigned genes. The least GO terms were assigned to the carbohydrate utilization, immune system process, and growth subclasses of the BP class; extracellular region part, virion, and virion part subclasses of the CC class; and translation regulator activity and protein folding chaperone subclasses of the MF class.

### 2.5. Transport and Metabolism of Carbon Sources

**Figure 5:**
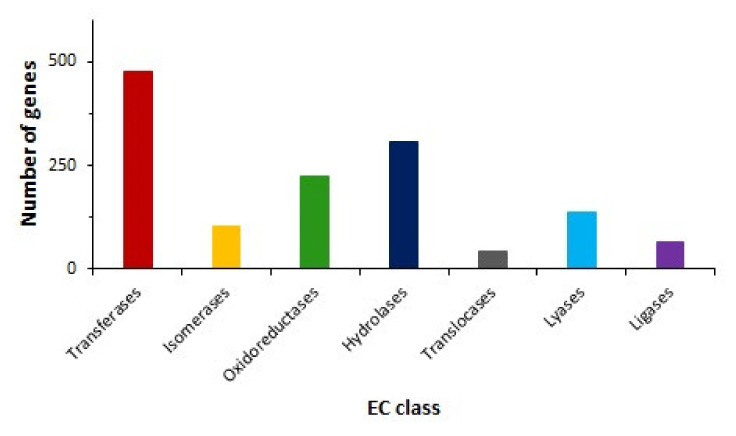
Distribution of genes encoding enzymes within enzyme class (EC) based on Kyoto Encyclopedia of Genes and Genomes (KEGG) pathways analysis. Colored bars indicate amounts of genes assigned to EC classes.

A distinctive capacity to utilize a broad spectrum of carbon sources, including monosaccharides, both pentoses and hexoses, disaccharides, and polyhydroxy alcohols, is one of the most significant features for industrial producers of bulk chemicals [2]. Biochemical tests based on the API 50CHE system revealed that the *Enterobacter* sp. LU1 strain has the ability to grow on a wide range of industrially relevant sugars, including glucose, fructose, lactose, sucrose, galactose, maltose, xylose, and cellobiose [1]. The ability to grow on these sugars was also demonstrated for the *K. aerogenes* LU2 strain in our previous study [17]. Analysis of LU1 strain genome showed that the largest group of C sources is transported via the phosphoenolpyruvate-dependent sugar phosphotransferase system (PTS), a major carbohydrate transport system in bacteria (Supplementary Table S2). The IIA, IIB, IIC, and IID components of PTS were recognized for glucose, fructose, sucrose, maltose, lactose, cellobiose, mannose, sorbose, and trehalose. Positive reactions in the API test have also been shown for many polyhydroxy alcohols, including d-mannitol and d-sorbitol, for which genes encoding specific PTS components were identified in the LU1 strain genome as well [1]. Furthermore, the PTS system was recognized for ascorbate, *N*,*N*’-diacetylchitobiose, *N*-acetylglucosamine, *N*-acetylgalactosamine, *N*-acetylmuramic acid, arbutin, and salicin. In turn, ribose, arabinose, and rhamnose, which also gave positive results in the API test, as well as galactose and xylose, are taken up by the ATP-binding cassette transporters (ABC transporters) [1,38]. Interestingly, maltose can be transported via the PTS system, but genes encoding ABC transporters for this disaccharide were also identified in the genome. Furthermore, genes encoding transporters belonging to the major facilitator superfamily (MFS) for arabitol, xylitol, and ribitol were recognized. Functional analysis at level 1 using the Kyoto Encyclopedia of Genes and Genomes (KEGG) database revealed that the percentage distribution of genes encoding enzymes within particular EC classes was very close to that described in the *K. aerogenes* LU2 (Figure 5) [17]. As with that strain, genes encoding hydrolases constitute one of the major groups in the LU1 strain genome, which can be related to the possibility of metabolizing a wide spectrum of C sources by this strain. These results indicate that *Enterobacter* sp. LU1 has a highly developed genetic background that allows a wide spectrum of C sources to be transported, which makes the strain an excellent candidate for industrial bio-based succinic acid production.

### 2.6. Glycerol Metabolism Pathways

Glycerol is among the main waste feedstock generated in large amounts during biodiesel production [39]. It was experimentally confirmed that this waste product can be used efficiently by LU1 strain as a cheap carbon source in SA production under anaerobic conditions [1,15]. Thus, the genetic background of glycerol metabolism under these conditions has been deeply investigated. The glycerol uptake facilitator was recognized as an important protein, which allows glycerol to be transported actively inside the cytoplasm [40]. In the LU1 strain genome, the *glpF* (FZF21_10700) gene coding for glycerol uptake facilitator was detected, according to the sequence data. Subsequently, intracellular glycerol is oxidized to dihydroxyacetone (DHA) and phosphorylated to dihydroxyacetone phosphate (DHAP). These reactions are catalyzed by glycerol dehydrogenase (GlyDH) and phosphoenolpyruvate (PEP)-dependent dihydroxyacetone kinase (DhaK) for which the presence of *gldA* (FZF21_10775) and *dhaK* (FZF21_12725) genes, respectively, was recognized in the genome. Afterwards, DHA is oxidized and converted into PEP, pyruvate (PYR), and acetyl-CoA [41]. PEP can be converted into oxaloacetate by phosphoenolpyruvate carboxykinase (PEPCK) encoded by *pckA* (FZF21_11895) gene, which was also detected, and then it can be reduced stepwise to succinate. In turn, pyruvate can be reduced to lactate due to the presence of *ldhA* (FZF21_18020) gene encoding lactate dehydrogenase (LDH), while acetyl-CoA can be transformed to acetate by both phosphate acetyltransferase (PTA) and acetate kinase (ACK) encoded by the *pta* (FZF21_17460) and *ackA* (FZF21_17465) genes, respectively, or reduced to ethanol by alcohol dehydrogenase (ADH) encoding by *adhE* (FZF21_20165) gene [42]. All of these genes have also been found in the LU1 strain genome, which explains the presence of byproducts such as lactate, acetate, and ethanol in the fermentation broth [1,15]. As reported by Yu et al. [39], in *E. coli*, there is another glycerol utilization pathway under anaerobic conditions. In this route, glycerol is phosphorylated to glycerol-3-phospate (G3P) by glycerol kinase and oxidized to DHAP by menaquinone-dependent G3P dehydrogenase (GlpABC). Importantly, in the LU1 strain genome, the *glpK* (FZF21_10695) gene for glycerol kinase and the *glpA* (FZF21_17665), *glpB* (FZF21_17660), and *glpC* (FZF21_17655) genes for anaerobic G3P dehydrogenase subunits A, B, and C, respectively, were also identified. These results indicate that *Enterobacter* sp. LU1 has a highly developed genetic background allowing for efficient utilization of glycerol. 

### 2.7. Metabolic Routes for SA Biosynthesis

Because *Enterobacter* sp. LU1 was recognized as a highly efficient SA producer, the genetic background of putative biochemical routes for succinate formation were investigated [1,15]. There are three possible biochemical succinate formation pathways: (1) the reductive branch of the TCA cycle, (2) the glyoxylate pathway, and (3) the oxidative TCA cycle [17]. Hence, the presence of genes involved in these pathways in the LU1 strain genome was evaluated. The reductive branch of the TCA cycle is used for SA formation under anaerobic conditions, where succinate plays a role as the H-acceptor instead of oxygen [43]. This biochemical route was reported for many *Klebsiella* strains, including *K. aerogenes* ATCC 29007, *K. aerogenes* AJ110637, and *K. aerogenes* LU2 [17,41,44]. In this pathway, the PEP is converted to succinate via intermediate compounds of TCA reductive branch cycle, including oxaloacetate, malate, and fumarate [43]. Based on the KEGG metabolic analysis, genes encoding phosphoenolpyruvate carboxylase (FZF21_10785), malate dehydrogenase (FZF21_12620), fumarate hydratase (FZF21_00650; FZF21_00325; FZF21_00320; FZF21_18775), and fumarate reductase (FZF21_07995; FZF21_07990; FZF21_07985; FZF21_07980) have been detected in the LU1 strain genome. Another potential biochemical route for succinate production is the glyoxylate pathway, where acetyl-CoA is converted to SA via citrate and isocitrate [43]. The KEGG map for glyoxylate and dicarboxylate metabolism reveals that genes encoding citrate synthase (FZF21_03400), aconitate hydratase (FZF21_20425; FZF21_06215), and isocitrate lyase (FZF21_08615) have also been recognized in the genome. The presence of genes involved in glyoxylate cycle is a great advantage of *Enterobacter* sp. LU1 strain, because during anaerobic culture and in the absence of additional electron donor, the activated glyoxylate route can contribute to achieving higher SA yield by providing extra NADH to the fermentative pathway [43]. Interestingly, the well-known SA producer *Actinobacillus succinogenes* 130Z (ATCC 55618) lacks the glyoxylate pathway in its genome, which shows the great potential of LU1 strain [45]. Genome analysis revealed that *Enterobacter* sp. LU1 also has a genetic basis for succinate production via the oxidative TCA cycle. In this route, succinate is formed from acetylo-CoA generated from pyruvate. However, under aerobic conditions, accumulation of SA in not naturally possible since it is an intermediate compound of the TCA cycle and is converted to fumarate by succinate dehydrogenase encoded by *sdhA* (FZF21_03385) gene. Therefore, in order to accumulate SA, it is necessary to block the *sdhA* gene, which will make converting succinate to fumarate impossible. These results show that LU1 strain has a genetic predisposition toward efficient succinate production. 

### 2.8. Dicarboxylic Acid Transporters

Under aerobic conditions, succinate present in the environment can be a source of carbon and energy for facultative anaerobic bacteria such as *Enterobacter* sp. LU1. However, under anaerobic conditions, it can also be the end product of fermentation and is thus excreted into the culture medium. As an efficient producer of SA, it is worth noting that genome analysis of LU1 strain indicated the presence of several genes encoding dicarboxylate transporters, including *dauA*, *dcuA*, *dcuB*, *dcuC*, and *dctA* genes. 

The *dauA* (FZF21_19960) gene encoding C4-dicarboxylic acid transporter DauA is involved in the aerobic transport of succinate from the periplasm to the cytoplasm under acidic conditions. Furthermore, it can play a role in the regulation of C4-dicarboxylic acid metabolism at neutral pH, by regulating the expression and/or activity of the *dctA* (FZF21_11275) gene encoding aerobic C4-dicarboxylate transport protein, which belongs to the dicarboxylate/amino acid:cation symporter family. This gene is responsible for the aerobic transport of fumarate, malate, and, to a lesser extent, succinate from the periplasm across the inner membrane. As reported by Valentini et al. [46], inactivation of the *dctA* gene in *Pseudomonas aeruginosa* PAO1 caused a growth defect of the strain in medium supplemented with succinate, malate, or fumarate, confirming the great importance of this gene in the C4-dicarboxylate transport system. 

Under anaerobic conditions, SA is excreted into the growth medium by DcuA and DcuB, anaerobic C4-dicarboxylate transporters belonging to the C4-dicarboxylate uptake family (Dcu). These transporters are encoded by *dcuA* and *dcuB* genes that have been identified in the FZF21_08090 and FZF21_19130 loci, respectively, and can operate as fumarate/succinate antiporters, excreting the latter [47]. As reported by Ullmann et al. [48], DcuA and DcuB can also transport Na^+^ in symport with dicarboxylates to avoid dissipating the proton motive force. The genome analysis also indicated the presence of the *dcuC* (FZF21_03800) gene encoding DcuC transporter belonging to a separate family of the C4-dicarboxylate efflux system. This carrier acts as a proton/succinate coexporter and plays a role in succinate excretion during fermentation [47,49]. Genes encoding DcuA, B, and C transporters have also been identified in the genome of another well-known SA-producer, *A. succinogenes* 130Z [45]. Furthermore, *dcuR* (FZF21_18795) and *dcuS* (FZF21_18800) genes, members of the two-component regulatory system DcuR/DcuS, involved in the C4-dicarboxylate-stimulated regulation of genes encoding the anaerobic fumarate respiratory system, were recognized in the genome. The results obtained indicate that the LU1 strain has the genetic predisposition to allow for efficient excretion of SA into the culture medium.

### 2.9. Osmotic and Oxidative Stress

It has been experimentally confirmed that *Enterobacter* sp. LU1 has the ability to grow with high concentrations of glycerol and lactose in the culture medium [1]. It proves that the strain is resistant to high osmotic pressure, which is a very important feature of industrial strains, allowing efficient batch fermentations to be conducted. Exploration of the LU1 genome has provided information about the presence of several genes involved in osmoregulation, such as the *aqpZ* (FZF21_02510) gene encoding aquaporin Z, the *osmY* (FZF21_06890) gene for osmotically inducible protein Y, and the *osmE* (FZF21_01050) and *osmB* (FZF21_20460) genes for osmotically inducible lipoprotein OsmE and OsmB, respectively. Furthermore, the *yehZYXW* operon encoding the osmoprotectant uptake system of the ABC transporter family and *OsmYXVW* genes, osmoprotectant ABC transporters, have also been identified in the genome. The analysis indicated that LU1 strain has many genes involved in osmoregulation, making it possible to carry out fermentation with high concentrations of carbon sources.

As we mentioned above, *Enterobacter* sp. LU1 has the ability to produce SA from glycerol and whey permeate under anaerobic conditions. In turn, under semi-aerobic conditions it is possible to produce SA using glycerol alone. Moreover, it has been shown that aeration at an early stage of strain culturing resulted in faster growth of bacterial biomass [15]. This is an important feature, especially considering that many industrial strains are strictly anaerobic microorganisms that require rich culture media and the preservation of stringent anaerobic conditions during biomass growth and fermentation [17]. Genome analysis indicated that LU1 strain has many genes involved in aerobic respiration and antioxidative stress, including the *sodA* (FZF21_10615), *sodB* (FZF21_00530), and *sodC* (FZF21_00480) genes encoding superoxide dismutase [Mn], [Fe], and [Cu-Zn], respectively, which participate in the destruction of superoxide anion radicals. Moreover, *katG* (FZF21_10770) gene for bifunctional enzyme with both catalase and peroxidase activity, *katE* (FZF21_01015) gene encoding catalase HPII, and gene (FZF21_00900) encoding glutathione peroxidase have also been detected. Importantly, genes for superoxide response transcriptional regulator SoxS and redox-sensitive transcriptional activator SoxR involved in control of the superoxide response regulon were recognized in the FZF21_08380 and FZF21_08375 loci. The analysis confirmed that *Enterobacter* sp. LU1 is an attractive microorganism for an industrial-scale applications due to its genetic background, allowing for growth and SA production under both anaerobic and semi-anaerobic conditions.

### 2.10. Horizontal Gene Transfer and Acquired Antimicrobial Resistance Genes

Genomic islands (GIs) are large genomic regions of probable horizontal origin. These regions often contain genes that can enhance the adaptability and competitiveness of microorganisms in a specific ecological niche, but they can also be related to antibiotic resistance or virulence [50]. Therefore, to evaluate the genetic diversity of *Enterobacter* sp. LU1, identification of GIs acquired through HGT was carried out using the IslandViewer 4 platform and the SIGI-HMM, IslandPick, IslandPath-DIMOB, and Islander prediction methods [50]. A total of 45 GIs with a length between 4029 bp and 65,642 bp were predicted in the genome of *Enterobacter* sp. LU1, with 20 by SIHI-HMM (187 protein products), 18 by IslandPick (174 protein products), and 7 by IslandPath-DIMOB (226 protein products) (Additional file 1). Manual inspection of particular GIs showed the presence of many genes encoding transcriptional regulators, but many phage-associated genes were also identified, confirming the results obtained using the Phaster platform. Interestingly, one *ugpC* gene encoding sn-glycerol-3 phosphate import ATP-binding protein UgpC, part of the ABC transporter complex ugpABCE involved in sn-glycerol-3-phosphate import, was identified within the acquired GIs. Moreover, four genes involved in type VI secretion system (T6SS) were recognized between FZF21_13895 and FZF21_13880 loci. As reported by Liu et al. [51], T6SS is widely distributed in gram-negative bacteria and was previously thought to be a factor related to virulence in animals and humans. However, recent studies indicate that T6SS is involved in microbial interaction and fitness in microflora [51]. Importantly, no virulence factors or homologs of virulence factors and no resistance genes or homologs of resistance genes were identified. Moreover, no pathogen-associated genes were recognized within the predicted GIs. The results obtained confirm the lack of acquired virulence and imported antibiotic-resistance genes in the LU1 strain genome, which is crucial in terms of using the strain as an industrial producer of SA. Due to the fact that ECC contains many bacterial species of both environmental and clinical importance, some of them may have innate resistance to a wide spectrum of antibiotics [22]. Therefore, exploration of the LU1 strain genome for the presence of antibiotic resistance-related genes using the ResFinder 3.2 platform was undertaken against “all databases,” with the selected identity (ID%) threshold and minimum length at the level of 98% and 80%, respectively [17,52]. The analysis showed that none of the resistance genes for aminoglycoside, macrolide, sulphonamide, fosfomycin, phenicol, quinolone, nitroimidazole, tetracycline, colistin, rifampicin, glycopeptide, fusidic acid, trimethoprim, or oxazolidinone were identified. However, one resistance *bla*_ACT-7_ (FZF21_08005) gene with predicted phenotype of beta-lactam resistant AmpC-type was recognized in the LU1 strain genome. To confirm the lack of resistance to the antibiotics mentioned above, further experimental studies of *Enterobacter* sp. LU1 will be carried out.

### 2.11. Prophage

Any bacterial strain can be infected with a virulent phage or contain one or more prophage sequences that were previously inserted and integrated into the bacterial chromosome or that exist as an extrachromosomal plasmid [53]. The presence of a prophage in the host genome has great biotechnological relevance for two reasons. On the one hand, it raises the feasibility of using phage-based genetic engineering. On the other hand, it suggests that the strain may be susceptible to phage lysis during industrial bioreactor processes [45]. Hence, genome exploration for the identification of putative prophage sequences was carried out. Three intact prophage regions in the LU1 strain genome were identified (Figure 1, Table 3, Supplementary Materials). In total, these regions represent 3.04% of the entire genome size. Within the largest intact prophage sequence (70.3 kb) encoded in the FZF21_04290-04755 region, of all 67 ORFs predicted, 56 and 11 were classified as phage and hypothetical proteins, respectively, using the Phaster platform [53]. In turn, for prophage sequence 2 (20.9 kb) encoded in the FZF21_21615-21740 region and prophage sequence 3 (49.8 kb) encoded in the FZF21_15580-15900 region, 24 and 38 phage proteins were identified, respectively. No bacterial proteins were found in any of the analyzed prophage regions. The highest number of proteins in a phage most similar to those in region 1 was found for *Cronobacter* phage ENT47670 (NC_019927), according to an analysis by the Phaster platform, with 20.9% protein similarity. In turn, for prophage region 2, the highest protein similarity (58.6%) was found for *Salmonella* phage 118970_sal3 (NC_031940), while *Enterobacteria* phage mEp235 (NC_019708) was the first hit for prophage region 3, with a protein similarity of 16%. The presence of a prophage is common in many bacterial strains, including SA-producers. McKinlay et al. [45] reported that in the genome of A. *succinogenes* type strain 130Z (ATCC 55618), a 39,489 bp prophage genome is encoded and the prophage organization is the most similar to *Aggregatibacter actinomycetemcomitans* phage AaΦ23. Moreover, one intact prophage region of length ~31.9 kb was also identified in the genome of *K. aerogenes* LU2, and the highest protein and nucleotide similarity was recognized for *Salmonella* phage RE-2010 (HM770079) and *Salmonella* virus Fels2 (NC_010463), respectively [17]. Importantly, the occurrence of a prophage in the bacterial genome may increase resistance to environmental stresses and support horizontal gene transfer (HGT), which contributes to increased biodiversity [54]. Thus, due to the fact that *Enterobacter* sp. LU1 was isolated from goat rumen, these features caused by the presence of prophage may provide benefits to the bacterial host. However, further studies on the effects of prophages on the physiology of the host strain should be carried out, and if the effects are negative, steps should be taken to eliminate these prophages from the host genome.

### 2.12. CRISPR

During the microbiological production of various metabolites at industrial scale, bacteriophage invasion can be a major issue [55]. Clustered regularly interspaced short palindromic repeats (CRISPR)/CRISPR-associated proteins (Cas) is an RNA-mediated, adaptive immunological defense mechanism that protects bacteria against foreign genetic elements, including phages or plasmids [56]. Therefore, the presence, frequency, and distribution of the CRISPR/Cas system within the LU1 strain genome were investigated using the CRISPRCasFinder platform [57]. Three CRISPR candidates with lengths of 105 bp, 931 bp, and 809 bp consisting of 1, 15, and 12 spacers (32–38 nt in size), respectively, were identified (Figure 1) (Table 4). CRISPR array 1 was excluded from further study due to low evidence level (value = 1) and designated as questionable. The annotation made by NCBI Prokaryotic Genome Annotation Pipeline (PGAP) confirmed the presence of CRISPR array 2 located between FZF21_024765 and FZF21_02470 loci and CRISPR array 3, which was found between FZF21_02435 and FZF21_02440 loci in the genome of *Enterobacter* sp. LU1. Furthermore, genes encoding I-F CRISPR-associated endonuclease Cas1 (FZF21_02465), I-F CRISPR-associated protein Csy2 (FZF21_02460), I-F CRISPR-associated protein Csy3 (FZF21_02455), and I-F CRISPR-associated endoribonuclease Cas6/Csy4 (FZF21_02450) were also recognized in the LU1 strain genome. In addition, a set of five irregularly distributed Cas genes was predicted, according to an analysis using the CRISPRCasFinder platform. Based on annotation provided by the NCBI PGAP, these genes were recognized as *recG* gene (FZF21_09460) encoding ATP-dependent DNA helicase RecG, *mdf* gene (FZF21_01425) encoding transcription-repair coupling factor, *dbpA* gene (FZF21_20690) encoding ATP-dependent RNA helicase DbpA, *srmB* gene (FZF21_16140) encoding ATP-dependent RNA helicase SrmB, and unnamed gene (FZF21_13020) encoding DEAD/DEAH family ATP-dependent RNA helicase. The results obtained indicate that *Enterobacter* sp. LU1 has a genetic defense mechanism against phage attack or invasion by foreign DNA, which is an important feature of this strain, especially since the CRISPR/Cas system is rarely found in the *Enterobacter* genus [17,58].

## 3. Conclusions

A hybrid approach in which Illumina short reads were combined with long reads generated by the ONT MinION platform enabled us to obtain a complete genome sequence with high accuracy in the form of one contig. The genome of *Enterobacter* sp. LU1 is one of 44 complete genomes of *E. hormaechei* that are sequenced to date, but it is the first sequence that has been fully characterized from a biotechnological perspective. Several desirable traits that are relevant to the potential use of *Enterobacter* sp. LU1 as an industrial strain were discovered at the genome level. Genes involved in transport and metabolism of carbohydrates and amino acids as well as the transcription process constitute the major group of genes. Genes associated with transport and metabolism of many industrially attractive carbon sources, including glycerol, have been identified in the genome. It has been shown that *Enterobacter* sp. LU1 has a genetic background suitable for the production of SA as the end product of fermentation via the reductive branch of the TCA cycle and the glyoxylate cycle. Furthermore, the mechanism of succinate transport has been deeply investigated and genes encoding dicarboxylate transporters have been identified in the genome. All of these findings may contribute to the high efficiency of succinate production and its secretion into the culture medium, and thus show considerable industrial potential. Moreover, many genes involved in the response to osmotic and oxidative stress have been recognized in the genome, which may explain the ability of LU1 strain to grow with high concentrations of carbon sources and under aerobic and anaerobic conditions. Due to the identification of prophage sequences, further studies into their effects on host strain physiology need to be performed, especially in terms of their impact on the fermentation process. Interestingly, rarely found CRISPR elements in the genus *Enterobacter* have been identified in the LU1 strain genome, and their role in protecting cells from infection needs to be verified. Importantly, the lack of acquired virulence or antibiotic resistance of the LU1 strain has been confirmed, which is crucial for industrial production of bulk chemicals. We believe that sequencing of the *Enterobacter* sp. LU1 genome will facilitate further exploitation of *Enterobacter* strains for biotechnology applications. In addition, the results presented here will enable transcriptome analysis, providing insight into the metabolic activity of LU1 strain. 

## 4. Materials and Methods

### 4.1. Strain

*Enterobacter* sp. LU1 was isolated from goat rumen and deposited in the International Culture Collection of Industrial Microorganisms (CCIM) at the Institute of Agricultural and Food Biotechnology under identification code KKP 2050p [1]. In previous studies, the strain was recognized as an efficient SA producer when crude glycerol and whey permeate served as carbon sources [1,15]. 

### 4.2. Growth Conditions

The LU1 strain was cultivated anaerobically in Oxoid 2.5 L jars (Thermo Fisher Scientific, Waltham, MA, USA) with Oxoid AnaeroGen 2.5 L sachets (Thermo Fisher Scientific, Waltham, MA, USA) in brain heart infusion (BHI) medium (Oxoid, UK) with the following composition (g/L): brain infusion solids: 12.5; beef heart infusion solids: 5.0; proteose peptone: 10.0; glucose: 2.0; sodium chloride: 5.0; disodium phosphate: 2.5; and pH = 7.4 at 37 °C for 16 h [17].

### 4.3. Genomic DNA Extraction

For quality assurance, genomic DNA was extracted and purified from a pure culture of a single bacterial isolate of *Enterobacter* sp. LU1 using the Genomic Micro AX Bacteria+Gravity kit (A&A Biotechnology, Gdynia, Poland) as per the manufacturer’s protocol (2017). The quantity and quality of extracted DNA were assessed by NanoDrop 2000 spectrophotometer (Thermo Fisher Scientific, Waltham, MA, USA) by measuring the absorbance at 230, 260, and 280 nm. Because long DNA fragments were required for ONT MinION, quality assessment of isolated DNA was also carried out by electrophoresis in 0.6% (*w*/*v*) agarose gel (EURx, Gdańsk, Poland) with SimplySafe dye (EURx, Gdańsk, Poland). DNA was visualized under UV light and archived using GelDoc XR+ gel documentation system and ImageLab software (Bio-Rad, Hercules, CA, USA). The size of the genomic DNA was compared to the Lambda DNA/*Hind*III molecular weight marker (Thermo Fisher Scientific, Waltham, MA, USA). Only high-quality and molecular weight samples (A260/280 = 1.8–2.0; >5 µg; >20 kb) were used to construct the fragment libraries. 

### 4.4. Re-Identification of Enterobacter sp. LU1

Prior to DNA sequencing, re-identification of LU1 strain using MALDI-TOF/MS (Bruker, Billerica, MA, USA) analysis paired with 16S rDNA sequencing has been carried out to exclude any risk of potential contamination at earlier stages of analysis. Mass spectra were compared with references in MALDI Biotyper 3.1 (Bruker, Billerica, MA, USA) using FlexControl software (Bruker, Billerica, MA, USA), while 16S rDNA sequence obtained was compared against sequences deposited in the NCBI GenBank using the NCBI BLAST algorithm [59].

### 4.5. Hybrid Whole-Genome Sequencing

Genome sequencing of LU1 strain was performed using the Illumina MiSeq platform and ONT MinION sequencer at Genomed SA. In brief, a paired-end library was prepared using NEB-Next DNA Library Prep Master Mix Set for Illumina (NEB, Ipswich, MA, USA) and then sequenced using Illumina MiSeq technology with 2 × 250 paired-end sequencing chemistry (Illumina, San Diego, CA, USA). For long-read sequencing by ONT MinION, a 1D long read library was constructed using the Ligation Sequencing Kit 1D and Native Barcoding Expansion 1–12 PCR-free (Oxford Nanopore Technologies, Oxford, UK). Subsequently, purified DNA was loaded onto the Flow Cell Mk 1 Spot-ON of the MinION sequencer using Library Loading Bead Kit R9 Version (Oxford Nanopore Technologies, Oxford, UK) following the manufacturer’s instructions, and sequencing was carried out for 24 h. After run completion, the raw reads were obtained using MinKNOW v. 1.7.14 (Oxford Nanopore Technologies, Oxford, UK) and base-calling of resulting data was performed using Albacore Sequencing Pipeline software (Oxford Nanopore Technologies, Oxford, UK).

### 4.6. De novo Assembly and Complete Genome Annotation

Raw reads were trimmed and quality filtering was performed using Cutadapt v. 1.9.1 [60]. De novo sequence data assembly from both sequencing platforms was conducted using Unicycler v. 0.4.7 software [31]. The genome was annotated by the NCBI PGAP with the best-placed reference protein set and GeneMarkS-2+ annotation methods [61].

### 4.7. Data Deposition

The genome sequence was deposited in the NCBI GenBank database in BioProject no. PRJNA562627 under accession number CP043438.

### 4.8. Phylogenomic Classification of Enterobacter sp. LU1

The genome sequence data were uploaded to the Type (Strain) Genome Server (TYGS) [23] for whole-genome-based taxonomic analysis [24]. Closest type strain genomes was determined in two complementary ways: First, the LU1 strain genome was compared to all type strain genomes available in the TYGS database via the MASH algorithm, a fast approximation of intergenomic relatedness [62], and the 10 type strains with the smallest MASH distances were chosen per LU1 strain genome. Second, an additional set of 10 closely related type strains was determined via the 16S rDNA gene sequences. These were extracted from the LU1 strain genome using RNAmmer [63], and each sequence was subsequently BLASTed [64] against the 16S rDNA gene sequence of each of the 11,252 type strains currently available in the TYGS database. This was used as a proxy to find the 50 best-matching type strains (according to bitscore) for the LU1 strain genome and subsequently calculate precise distances using the Genome BLAST Distance Phylogeny (GBDP) approach under the “coverage” algorithm and distance formula *d*_5_ [24]. These distances were finally used to determine the 10 closest type strain genomes to the LU1 strain genome. All pairwise comparisons among the set of genomes were conducted using GBDP and accurate intergenomic distances inferred under the “trimming” algorithm and distance formula *d*_5_ [24]. One hundred distance replicates were calculated for each. Digital DDH values and confidence intervals were calculated using the recommended settings of GGDC 2.1 [24]. The resulting intergenomic distances were used to infer a balanced minimum evolution tree with branch support via FASTME 2.1.4 including Subtree Pruning and Regrafting (SPR) postprocessing [29]. Branch support was inferred from 100 pseudo-bootstrap replicates. The trees were rooted at the midpoint [30] and visualized with PhyD3 [65]. Type-based species clustering using a 70% dDDH radius around each of the 14 type strains was done as previously described [24]. Subspecies clustering was done using a 79% dDDH threshold as previously introduced [24]. Furthermore, Orthologous Average Nucleotide Identity Tool (OAT) software [27] was used for calculation of OrthoANI values between strains belonging to the ECC. 

### 4.9. Functional Classification of Annotated Genes and Other Genome Analysis

RNAmmer 1.2 and tRNAscan-SE 2.0 were employed to predict rRNAs and tRNA-encoding genes, respectively [63,66]. Functional analysis by Clusters of Orthologous Groups of proteins (COGs) and Gene Ontology (GO) annotation were done by using the eggNOG 4.5 orthology prediction pipeline [67]. Genes encoding enzymes were predicted with the Kyoto Encyclopedia of Genes and Genomes (KEGG) database and definitions of KEGG identifiers were collected with TogoWS [68,69]. The genes involved in transport and metabolism of carbon sources as well as transport of dicarboxylic acids were manually searched. Genes encoding enzymes involved in osmotic and oxidative stress were identified with Rapid Annotations using Subsystems Technology (RAST) [70]. Genomic islands (GIs) were determined using IslandViewer [50] and antibiotic resistance-related genes were predicted using ResFinder 3.1 [52]. Annotation of prophage sequences within the genome was performed with Phage Search Toll Enhanced Release (Phaster) [53]. The presence of CRISPR/Cas system was checked using CRISPRCasFinder [57]. The whole-genome sequence was visualized with CGView software [71]. 

## Figures and Tables

**Table 1:** General information on genome sequencing project of *Enterobacter* sp. LU1.

Properties	
Strain	*Enterobacter* sp. LU1
Sample source	Goat rumen
Sequencing type	Hybrid (short/long read sequencing)
Sequencing platforms	Illumina MiSeq/ONT MinION
Library type	1 library with 400 bp insert/1D long read library
Fold average coverage	98 ×/150 ×
Assembly method	Unicycler v. 0.4.7
Assembly level	Complete genome
Annotation method	Best-placed reference protein set; GeneMarkS-2+
Annotation pipeline	NCBI Prokaryotic Genome Annotation Pipeline
BioProject	PRJNA562627
BioSample	SAMN1264612
Accession number	CP043438

**Table 2:** General features of *Enterobacter* sp. LU1 genome compared to *K. aerogenes* LU2 (formerly *E. aerogenes*) genome and 15 complete *Enterobacteriaceae* genomes ^a^.

Genome Features	*Enterobacter* sp. LU1	*K. aerogenes* LU2	*Enterobacteriaceae* Average ^b^	*Enterobacteriaceae* Range ^b^
Genome size (bp)	4,636,526	5,0626,51	5,133,067	4,641,652–5,452,368
G+C (%)	55.6	55	53.3	50.6–57.5
No. of contigs	1	1	2.5	1–10
No. of plasmids	0	0	1.5	0–9
Total genes	4425	4986	5045	4532–5523
Total CDSs	4311	4868	–	–
Protein-coding genes	4283	4741	4804	4242–5300
Gene density (gene/kb)	0.954	0.985	0.983	0.951–1.082
5S rRNAs	9	8	24	19–25
16S rRNAs	8	7		
23S rRNAs	8	7		
tRNAs	84	86	84	76–88
ncRNAs	5	10	–	–
Pseudogenes	49	127	115	1–205
CRISPR arrays	2	0	–	–
Prophages	3	1	–	–

“–” no data available. ^a^ *Enterobacter cloacae* PIMB10EC27, *E. cloacae* subsp. cloacae ATCC13047, *Enterobacter hormaechei* C45, *E. hormaechei* subsp. *steigerwaltii* DSM 16691, *Klebsiella aerogenes* G7, CAV1320 strains, *Raoultella ornithinolytica* A14, S12, B6 strains, *Citrobacter freundii* CAV1321, FDAARGOS73, CFNIH1 strains, *Escherichia coli* NRG857c, K12, AIA39 strains. ^b^ Data based on genome summary pages of NCBI GenBank database.

**Table 3:** Intact prophage regions identified in genome of *Enterobacter* sp. LU1.

Region	Region Length (kb)	Completeness	Total Proteins	Region Position	GC (%)
1	70.3	Intact	67	1,116,715–1,187,045	52.96
2	20.9	Intact	29	2,196,837–2,217,799	52.75
3	49.8	Intact	50	3,424,122–3,473,981	50.02

**Table 4:** Distribution of clustered regularly interspaced short palindromic repeats (CRISPR) regions in genome of *Enterobacter* sp. LU1.

Region	Start	End	Spacers	Repeat Consensus/Cas Genes	Evidence Level
1	1,143,757	1,143,850	1	TGTCCAGCCGATGTCCAGCCAGTGTCCA	1
2	1,566,815	1,567,745	15	GTTCACTGCCGTGCAGGCAGCTTAGAAA	4
3	1,571,432	1,572,180	12	GTGCACTGCCGTACAGGCAGCTTAGAAA	4

## Supplementary Materials

Supplementary Materials can be found at https://www.mdpi.com/1422-0067/21/14/4835/s1.
